# The Role of Extracellular Vesicles in Epstein–Barr Virus Immunology and Pathogenesis for Possible Diagnostic and Therapeutic Direction

**DOI:** 10.1155/cjid/4451657

**Published:** 2026-09-24

**Authors:** Raha Safari Roudsari, Nazanin Bayat, Mehdi Shabani, Razieh Dowran

**Affiliations:** ^1^ Department of Medical Laboratory Sciences, School of Allied Medical Sciences, Tehran University of Medical Sciences, Tehran, Iran, tums.ac.ir; ^2^ Department of Medical Biotechnology, Faculty of Medical Sciences, Tarbiat Modares University, Tehran, Iran, modares.ac.ir; ^3^ Department of Virology, School of Public Health, Tehran University of Medical Sciences, Tehran, Iran, tums.ac.ir; ^4^ Research Center for Clinical Virology, Tehran University of Medical Science, Tehran, Iran, tums.ac.ir

## Abstract

Epstein–Barr virus (EBV), a member of the herpesvirus family, is extremely prevalent worldwide. EBV is a double‐stranded DNA virus associated with various malignancies, autoimmune diseases, and lymphomas. Transmission occurs through different means, including saliva and organ transplantation. EBV can produce and secrete vesicles that are critical in intracellular communication and valuable biomarkers in body fluids to diagnose potential diseases. They can also be utilized in targeted therapy. Exosomes are extracellular vesicles (EVs) that play a significant role in several biological processes. Some important components in EBV‐related EVs can be valuable biomarkers, including proteins such as latent membrane protein 1 (LMP1) and LMP2A, microRNAs (miRNAs), and long noncoding RNAs (lncRNAs). This review explores the diagnostic and therapeutic potential of EBV‐derived exosomes. It also highlights their challenges due to the lack of standardization and difficulties in exosome isolation and improving exosome‐based therapy efficiencies.

## 1. Introduction

**FIGURE 1:**
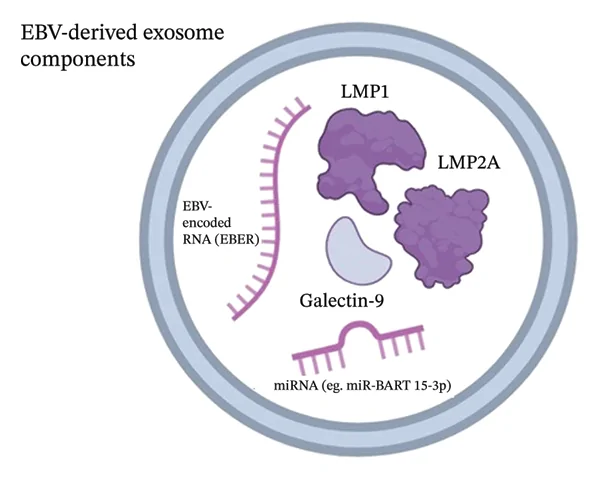
Schematic diagram showing the important components of EBV‐derived exosomes.

Epstein–Barr virus (EBV) is well known among the herpesvirus family. EBV is very prevalent among adults, and up to 90% of the worldwide population has been infected with EBV. EBV is a double‐stranded linear DNA virus known for causing B‐cell lymphoma. This linear DNA is enclosed with glycoproteins that can assist in the entrance of the genome into the host B cell. EBV exists in two primary phases: latent and lytic. In the latent phase, the replication is inactive and only restricted genes are expressed. These genes are vital for the maintenance of the virus in a dormant state [1]. The latent genes expressed by the EBV genome in lymphoblastoid cell lines are as follows: EBV nuclear antigens (EBNA1, 2, 3A, 3B, 3C, and the EBNA leader protein [EBNA‐LP]), latent membrane proteins (LMP1 and LMP2), and EBV‐encoded small RNAs (EBER1 and EBER2) and miRNAs [2] (Figure 1). In contrast, in the lytic phase, active replication occurs, which leads to infectious virion production. The lytic phase contributes to oncogenesis by producing infectious particles and regulating cellular processes [3]. Through their viral genome replication, they can cause the differentiation of B cells into memory B cells and then transformation into malignant B‐cell lymphoma [4].

**FIGURE 2:**
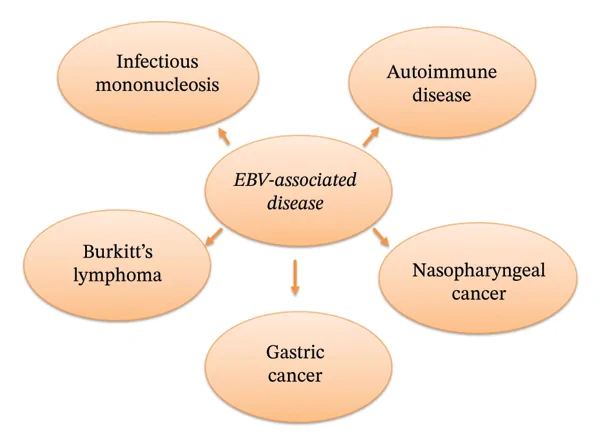
Examples of EBV‐related diseases. Epstein–Barr virus (EBV) infection is linked to various malignancies, including B‐cell lymphoma, nasopharyngeal carcinoma (NPC), gastric cancer, Burkitt’s lymphoma (BL), and autoimmune diseases such as multiple sclerosis (MS) and infectious mononucleosis.

Besides B‐cell lymphomas, EBV is also associated with a wide range of other malignant and noninvasive diseases, including nasopharyngeal carcinoma (NPC), gastric cancer, and hemophagocytic syndrome. Burkitt’s lymphoma (BL), which is common in malaria‐endemic regions, is also the result of EBV infection [5]. It has been shown that the EBNA1 gene of EBV can inhibit apoptosis and lead to fast‐growing B cells through interacting with p53‐regulators and antiapoptotic proteins. Infectious mononucleosis (IM) is another common disease related to EBV with symptoms such as fatigue, fever, and lymphadenopathy [6]. As mentioned above, EBV can be transmitted by organ transplantation. This condition occurs in immunocompromised patients, which is known as post‐transplant lymphoproliferative disorder (PTLD). Autoimmune diseases are another category of disorders whose risk is increased by EBV. EBV acts as an important trigger that can induce immune intolerance. Several reports have reported an association between EBV infection and exacerbation of these diseases. Examples of autoimmune diseases are dermatomyositis, systemic lupus erythematosus (SLE), and rheumatoid arthritis [7] (Figure 2).

EVs are lipid bilayers with sizes ranging from 20 to 1000 nm. A variety of EVs exist, such as microvesicles, apoptotic bodies, and the most pivotal one, exosomes [8]. Exosomes are membrane‐contained EVs that carry different molecules, including nucleic acids, proteins, and lipids [9, 10]. In other words, exosomes are the subset of EVs distinguished by their endosomal origin and size, whereas the other category of EVs includes larger vesicles such as microvesicles and apoptotic bodies. The transportation of this cargo allows exosomes to carry out essential functions, such as clearance of cellular waste [10] and protection of their contents from degradation [11]. Exosomes also contribute to the preservation of homeostasis via transferring the key molecules between cells [9]. Multivesicular bodies (MVBs) contain exosomes and release them into the extracellular space when fused with the plasma membrane [12]. They incorporate diverse cargo, including nucleic acids, proteins, and lipids. Once matured, MVBs can undergo degradation by fusion with lysosomes or merge with the plasma membrane to secrete their intraluminal vesicles (ILVs), known as exosomes [9]. Exosomes are recognized as essential regulators of cell–cell communication, playing a role in physiological operations, e.g., immune responses and regeneration, and pathological processes such as cardiovascular diseases and inflammation [9, 13]. They can also lead to the promotion of tumors through various processes, such as preventing apoptosis, enhancing angiogenesis, and developing resistance to chemotherapy [9].

EBV produces several molecules, including proteins, nucleic acids, and miRNAs, whose presence in exosomes facilitates their fusion, communication between cells, and pathogenic functions. These viral components can induce numerous changes in signaling cascades in recipient cells. LMP1‐containing exosomes can suppress T‐cell proliferation and tumor cell evasion from the immune system [14]. Another vital component called LMP2A can activate NF‐kB, which is an oncogenic signaling pathway [15]. EBV‐encoded miRNAs play an essential role in modulating host and viral gene expression. Certain miRNAs, especially the BART family, are highly expressed in EBV‐derived exosomes in different tumors, such as gastric cancer and NPC. EVs released from EBV‐infected cells contain many important components that can stimulate the expression of various cytokines and chemokines, such as IL‐4, IL‐6, IL‐8, and IL‐13, which leads to the development of disease and the tumor microenvironment [16].

**FIGURE 3:**
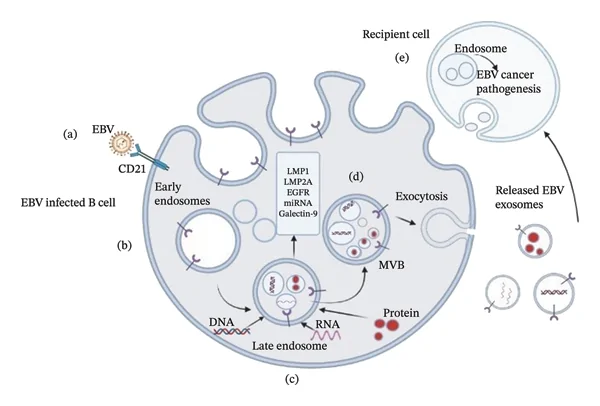
Schematic representation of exosome biogenesis in EBV‐infected cells. (a) EBV (Epstein–Barr virus) attaches to the CD21 receptor on the surface of a target B cell or epithelial cell. (b) Following internalization, early endosomes are formed. (c) Early endosomes mature into late endosomes. (d) Multivesicular bodies (MVBs) develop, and intraluminal vesicles (ILVs) are loaded with EBV‐derived cargo, including LMP1 (latent membrane protein 1), viral miRNA, and EBERs. (e) MVBs fuse with the plasma membrane, releasing exosomes into extravesicular space, delivering their pathogenic cargo to neighboring cells and promoting tumorigenesis.

The biogenesis of exosomes begins with the cargo endocytosis and early endosome formation that can convert to late endosomes or MVBs through maturation. Afterward, the cargo is sorted in ILVs, and then, the MVB membrane fuses with the plasma membrane and releases the ILV out of cells. After the release of EVs into the extracellular space, they can induce different types of impacts on neighboring cells. These effects include internalization, ligand–receptor interaction, and membrane fusion [17] (Figure 3).

EVs, especially exosomes, are not only responsible for viral communication but also play a pivotal role in both diagnosis and treatments. Early diagnosis of the disease leads to better patient outcomes and easier treatment. EBV‐related exosomes, which contain a large number of pathogenic factors, can also be a strong target for cancer diagnosis and therapy. This report aims to represent current findings about EBV‐related EVs and summarize the role of EVs and exosomes in EBV biology and highlight the advances and future of this field. However, important challenges remain unaddressed, including the specificity of candidate biomarkers due to their presence in noninfected cells and the failure of experimental results from cell culture models (in vitro) to translate into clinical settings (in vivo).

## 2. Biogenesis and Composition of EBV‐Derived Exosomes

### 2.1. Hijacking the Host Exosome Pathway

#### 2.1.1. How EBV Manipulates the Host Cell’s Exosome Biogenesis Machinery

In EBV‐infected cells, viruses can hijack the host’s endosomal system and metabolic pathways for their own assembly. They may alter the contents of EVs to transfer viral factors to uninfected cells, thereby promoting viral spread and contributing to disease progression [18, 19]. This demonstrates that both EVs and the virus share similar mechanisms for cell entry and biogenesis [20]. Then, EBV regulates cell‐to‐cell signaling by secreting viral components such as the oncoprotein LMP1 into EVs of the host cell. The incorporation of LMP1 into exosomes is correlated with an increase in the oncogenic potential of the secreted vesicles [21]. Moreover, key signaling pathways, such as PI3‐K/Akt, Wnt/β‐catenin, NF‐κB, and JAK/STAT, can be exploited by LMP1 to drive EBV‐associated disease progression and tumor development [22]. It has been observed in multiple studies that viruses can alter EV cargo to transfer viral components to uninfected cells, promoting viral spread and pathogenesis [18]. In addition, exosomes from EBV‐infected cells have been found to export host proteins that regulate the immune response. This process may serve as a strategy for the virus to evade the immune system. Some of the other effects of exosomes are as follows: Exosomes containing FasL from EBV‐infected cells have been shown to induce apoptosis in recipient cells by activating the extrinsic apoptotic pathway, involving caspase‐3, 7, and 8 [19]. On the other hand, exosomes released from EBV + B cells can result in increased interleukin‐10 (IL‐10) and programmed death‐ligand 1 (PD‐L1) expression by stimulating STAT3 phosphorylation in cutaneous T‐cell lymphoma (CTCL) cells. This may lead to immune evasion mechanisms in T/NK‐cell lymphoproliferative diseases [23].

### 2.2. Molecular Cargo of EBV‐Derived Exosomes

#### 2.2.1. Viral Components

EBV produces several molecules such as proteins and nucleic acids, which are incorporated into exosomes, facilitating intercellular communication; therefore, exosomes play a key role in the transport of these molecules and their protection from degradation. The sorting of molecular cargo, such as proteins and nucleic acids, into exosomal vesicles primarily occurs through ILVs formation. In this process, selected molecules are packaged within small vesicles that bud into the lumen of MVBs. Following the fusion of MVBs with the plasma membrane, the ILV maturation occurs. In order to sort proteins into exosomes, the ESCRT is necessary. LMP1 acts as an important oncoprotein of EBV and is incorporated into the membrane of exosomes. Exosomes containing LMP1 can suppress T cells. Moreover, following B‐cell infection, LMP1 results in the formation of lymphoblastoid cell lines [24]. Regarding nucleic acids, EBV holds a linear double‐stranded DNA that encodes more than 80 proteins [25] and also expresses both EBER1 and EBER2 and miRNAs [2]. Viral miRNAs can modulate viral transcripts and host gene networks, so that they contribute to the virus’s infectious cycle [26]. Besides leading to disease development [27] and cancer metastasis [28], EBV miRNAs trigger a range of downstream processes. For example, it has been observed that macrophage activity can be influenced by exosomes derived from EBV + lymphoma [24]. On the other hand, the *Bam*HI fragment A rightward transcript (BART) region consists of 22 miRNA precursors (BART1 to BART22), which generate 44 mature miRNAs. Different formations of long noncoding BARTs (lnc‐BARTs) are produced by alternative splicing of BARTs [28]. miR‐BARTs have been detected in exosomes secreted by epithelial cells with EBV infection and promote the growth of infected cells, thus disseminating the infection. Playing a key role in the correlated mechanisms, miR‐BARTs can contribute to metastasis and proliferation [24]. As an example, miR‐BART1‐3p can result in the improvement of gastric cancer via the exosome pathway [27]. Besides the mentioned viral components, EBV also has EBNA1, 2, 3A, 3B, and 3C and EBNA‐LP. EBNA1 contributes to the expression of LMP1, whereas EBNA2 and EBNA‐LP lead to the survival of B cells and naïve B cells with EBV infection, respectively. EBNA3, on the other hand, has been shown to result in cell proliferation by inhibiting the transcription of CDKN2B [2] (Table 1).

**TABLE 1:** Key components of EBV‐derived exosomes and EVs, their functions, and related diseases.

Component	Function	Related diseases	Detection methods/sample type
Latent membrane protein 1 (LMP1)	An important oncoprotein of EBV/suppresses T cells/forms lymphoblastoid cell lines	B‐cell lymphoma, nasopharyngeal carcinoma (NPC)	Western blot, ELISA/plasma cell culture supernatant
EBNA1, 2, 3A, 3B and 3C and EBNA‐LP	EBNA1 contributes to the expression of LMP1/EBNA2 and EBNA‐LP leads to the survival of B cells and naïve B cells/EBNA3 results in cell proliferation by inhibiting the transcription of CDKN2B	B‐cell lymphoma, NPC	RT‐qPCR, immunohistochemistry/tissue biopsy, serum
EBV‐encoded miRNAs (miR‐BARTs)	Promote the growth of infected cells/contribute to metastasis and proliferation	EBV‐associated gastric cancer (EBVaGC), hemophagocytic lymphohistiocytosis (HLH)	RT‐qPCR, RNA‐seq/plasma exosome palette

*Note:* NPC, nasopharyngeal carcinoma; HLH, hemophagocytic lymphohistiocytosis.

Abbreviation: EBVaGC, EBV‐associated gastric cancer.

#### 2.2.2. Host‐Derived Molecules

The viral miRNAs and proteins released from exosomes, which are secreted by cells with EBV infection, stimulate the expression of cytokines and chemokines by target cells so that the microenvironment changes in favor of the virus [29]. Genetics, environment, and age affect how the immune system responds to EBV infection [30]. Cytokines and chemokines such as IL‐4, IL‐6, IL‐8, IL‐13, CXCL10, and CXCL12 are overexpressed in EBV‐associated tumors and lead to the development of the disease [7]. EBV can decrease the signaling of interferon [31], and IFI16 (interferon gamma inducible protein 16) and cleaved interleukins contribute to host immune evasion, while IL‐1β is believed to support EBV endurance [29]. To maintain an immune‐suppressive microenvironment in EBV‐associated malignancies, IL‐10 downregulates HLA Class 1 and 2 antigen expression and inhibits CD8+ T‐cell cytotoxicity. EBV proteins also play an immunosuppressive role, with LMP1 and EBNA1 enhancing the expression of mediators. Additionally, EBV gastric cancer cells express more of CCL20, CCL22, and CCL17 compared to other EBV‐related tumors [7].

### 2.3. Techniques for Studying EBV‐Derived Exosomes

#### 2.3.1. Isolation and Characterization Methods

EVs function as a cellular messaging system and facilitate the conveyance of information between cells so that the organismal homeostasis is conserved [32]. As EVs are highly important and difficult to detect, it is essential to develop optical methods for their isolation and characterization to fully understand their translational applications [33, 34]. Centrifugation‐based methods are regarded as the most effective ones for isolating exosomes. This process allows the sedimentation of vesicles in solution [35]. Since EVs and viruses have low density, they can be pelleted from the solution by high‐speed centrifugation; therefore, ultracentrifugation has been used for decades as a fast and reliable method for isolating viruses and EVs. This technique is affordable, requires minimal technical skill, and enables the maintenance of esterase enzymatic activity in EVs [32, 33].

Flow cytometry is another known technique for rapid analysis and measurement of EVs. This method relies on the capture of fluorescence and light scattering from individual nanosized EVs suspended in a solution [35]. In addition, nanoscale flow cytometry, besides nanoFACS (vesicle flow cytometry), is utilized for distinguishing viruses from EVs, separating EV subpopulations from a diverse input population [32]. Furthermore, to analyze the RNA content of EVs, high‐throughput RNA‐Seq is used, confirmed by reverse transcriptase quantitative polymerase chain reaction (RT‐qPCR) [33]. Beyond their composition and biogenesis, EBV‐derived exosomes noticeably influence host immunology. The cargo composition of EBV exosomes largely dictates their immunological effects. Understanding how EBV exosomes are generated and loaded with specific cargo provides an essential framework for illuminating their roles in immunobiology. The following section clarifies their impact on immune response and other complications.

## 3. Role of EVs and Exosomes in EBV Immunobiology

### 3.1. Immune Evasion

#### 3.1.1. How EBV‐Derived Exosomes Modulate the Host Immune Response

**FIGURE 4:**
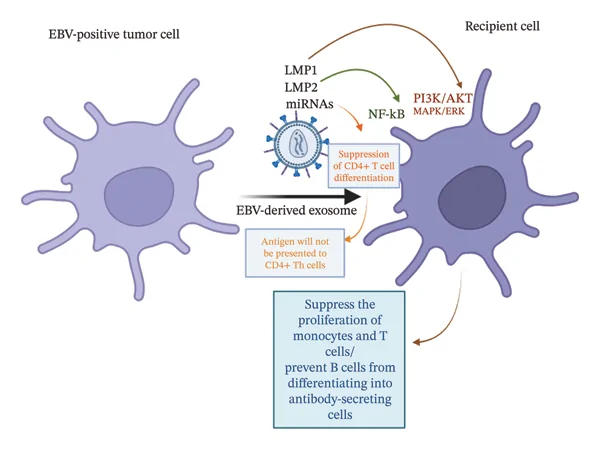
Molecular mechanisms of EBV exosome‐mediated immune modulation in the tumor microenvironment. EBV‐derived exosomes from tumor cells transfer LMP1, LMP2A, and viral miRNAs to recipient cells. LMP1 activates PI3K/AKT and MAPK/ERK signaling cascades and cell proliferation and prevents B‐cell differentiation. LMP2 activates NF‐Kb oncogenic pathway. Viral miRNAs suppress CD4+ T‐cell differentiation and prevent the combination of EBV antigen with MHC II molecules, thereby blocking antigen presentation to CD4+ helper T cells and facilitating immune evasion.

By carrying viral proteins and RNAs, EBV‐modified exosomes can initiate signaling cascades in recipient cells [36]. It has been observed that EBV‐positive cells secrete exosomes containing LMP1, which enables the tumor cells to evade the immune system by suppressing the proliferation of monocytes and T cells [37]. These exosomes initiate PI3K/AKT and MAPK/ERK pathways in recipient cells. LMP1 also suppresses immune responses by preventing B cells from differentiating into antibody‐secreting cells. Additionally, LMP2 can activate NF‐kB, which is an oncogenic signaling pathway. On the other hand, EBV miRNAs lead to a decrease in the activation of cytotoxic EBV‐specific CD4+ effector T cells due to the suppression of CD4+ T‐cell differentiation [36]. EBV miRNAs also prevent the combination of EBV antigen with MHC II; therefore, the antigen will not be presented to CD4+ Th cells [38]. BART miRNAs play a role in modulating TNF‐α, IL‐10, and ARG1 expression levels in EBV‐positive lymphoma cells [24]. In EBV‐positive nasopharyngeal cells, EBV miR‐BART22 represses the expression of LMP2A, so it is not recognized by CD4+ and CD8+ T cells anymore. The same applies to EBV miR‐BART9, EBV miR‐BART16, and EBV miR‐BART17‐5p and LMP1 expression [38] (Figure 4).

### 3.2. Viral Persistence and Latency

#### 3.2.1. Role of Exosomes in Maintaining EBV Latency and Facilitating Reactivation

The EBV infection cycle comprises two phases: the lytic (replicative) phase and the latent phase [39]. EBV exosomes contain miRNAs, which are effective in the pathobiology of the EBV life cycle. They are capable of regulating the transition between the latency and lytic phases of EBV by targeting host and viral mRNAs [40]. The expression pattern of EBV miRNAs is associated with the stage of viral latency [41]. LMP1 can strengthen the latency phase by suppressing the initiation of the lytic phase through the NF‐kB signaling pathway. Therefore, LMP1 plays a key role in modulating the maintenance of EBV latency [42].

### 3.3. Intercellular Communication

#### 3.3.1. How EBV Exosomes Facilitate Communication Between Infected and Uninfected Cells

Exosomes released by NPC cells with EBV infection can stimulate growth‐signaling pathways in uninfected cells, and then, this effect is amplified by LMP1. LMP1 and viral miRNA are carried to uninfected cells, and miRNAs selectively suppress their human miRNA targets [41].

#### 3.3.2. Transfer of Viral Components to Neighboring Cells

Exosomes are a vital cellular mechanism for transferring various molecules, including proteins and miRNAs, between cells. Regarding EBV, LMP1 and several miRNAs are secreted from exosomes, and they alter the behavior of nearby cells [43]. EBV exosomes carry their miRNAs, which influence mitochondrial respiration in target cells, so that metabolism is altered in the tumor microenvironment [44]. EBV exosomes can deliver viral components such as LMP1 to the tumor microenvironment and enhance cancer‐promoting signals in nearby cells. Liu et al. discovered that LMP1 was present in macrophages in the tumor microenvironment [45].

## 4. Pathogenic Roles of EBV‐Derived Exosomes and Exosomes in Diseases

### 4.1. EBV‐Associated Cancers

#### 4.1.1. NPC

##### 4.1.1.1. Role of Exosomal miRNAs in Tumor Progression

The diverse molecular cargo of EBV‐derived exosomes contributes to the extension of pathological complications and the development of several EBV‐associated cancers. In other words, the pathogenic activities of EBV‐derived exosomes arise from the specific molecular cargo they carry. Tumors associated with EBV develop through distinct mechanisms of tumorigenesis [24]. Multiple studies have shown that this virus is correlated with gastric, lymphoid, and epithelial malignancies, such as NPC [46–48]. More than 90% of patients diagnosed with undifferentiated NPC have EBV infection [49]. Yuan et al. demonstrated that EBV infection decreases the susceptibility of NPC cells to ferroptosis by activating the p62‐Keap1‐NRF2 signaling pathway while simultaneously increasing the expression of SLC7A11 and GPX4; high levels of GPX4 are associated with unfavorable clinical outcomes in patients with NPC [48]. Furthermore, in EBV‐associated NPC, tumor cells undergo lytic reactivation and lytic proteins of EBV have been observed to result in genome instability [50]. On the other hand, antigens produced by nasopharyngeal epithelial cells infected with EBV play distinct roles in NPC progression. For instance, EBNA1 and LMP1 lead to NPC progression by converting nasopharyngeal epithelial cells into NPC cells and stimulating cancer cell growth, respectively [47]. EBV encodes 44 miRNAs, which can be transferred to recipient cells by exosomes. These exosomes allow the constant transfer of various cargos between donor and recipient cells. This process facilitates intercellular communication [26, 51]. On the other hand, EBV miRNAs regulate viral transcripts, playing a role in the virus’s infectious cycle. EBV miRNAs fall into three groups, including BART Cluster 1, BART Cluster 2, and *Bam*HI fragment H rightward open reading frame‐1 (BHRF 1) [26]. It has been proven that EBV miR‐BART11 and EBV miR‐BART17 target FOXP1 and PBRM1, respectively, which inhibit the expression of PD‐L1. This leads to overexpression of PD‐L1 and therefore tumor immune evasion [51]. miR‐BARTs play a role in metastasis and tumor recurrence in NPC. For instance, BART13‐3p promotes metastasis by inducing epithelial–mesenchymal transition through downregulating the tumor suppressor AB12 [28]. Moreover, EBV miR‐BART10‐ 5p increases in NPC samples, thus acting as a diagnostic factor in NPC. The mentioned miRNA enhances angiogenesis by increasing vascular endothelial growth factor (VEGF) expression and restricting Spry3, which is a tumor suppressor. miR‐155 is delivered to retinal pigment epithelial cells by BL cells infected with EBV, contributing to an elevation in VEGF levels, which results in tumor angiogenesis [26].

##### 4.1.1.2. Lymphomas

In Hodgkin lymphoma, miR‐BARTs 19‐3p and 13‐3p are found, although miR‐BARTs in this lymphoma are expressed at lower levels than in other cancers. In addition, in NK/T‐cell lymphoma, there are high levels of BART19‐3p [28]. Higuchi et al. have shown that the BART Cluster 2 region makes a significant contribution to the formation of lymphoproliferative diseases [52].

##### 4.1.1.3. Exosomal Proteins and Their Oncogenic Effects

Several investigations have been conducted to discover the functions of viral proteins (e.g., EBNA1 and LMP1) in EBV‐associated cancers. These proteins organize the exosomes’ formation after being released into them [24, 48]. It has been found that EBV proteins contribute to the suppression of tumor cell apoptosis and the development of tumorigenesis. LMP1 is one of the mentioned proteins, interacting with human growth factor‐regulated tyrosine kinase substrate (Hrs) and Syntenin‐1, proteins that play a key role in guiding LMP1 into EVs for packaging and release. Exosomes containing LMP1 have immunosuppressive functions as they could suppress T cells. In addition, LMP1 downregulates miR‐203, which acts as a tumor suppressor [24]. On the other hand, EBNA1, the viral protein EBV transcription factor, stimulates demyelination, resulting in deterioration of multiple sclerosis (MS) [25].

##### 4.1.1.4. Gastric Carcinoma

Gastric cancer is another epithelial malignancy associated with EBV [49]. In this cancer, stomach epithelial cells with EBV infection undergo clonal growth. DNA methylation of CpG islands is detected in the promoter regions of several cancer‐related genes in EBVaGC, and then, clonal cell growth emerges, followed by inflammation‐associated signals and genomic mutations in cancer cells. Consequently, tumor cells change the constituent cells of their microenvironment. Finally, these phenomena lead to the progression of the tumor [46].

##### 4.1.1.5. Contribution of EBV Exosomes to Tumor Microenvironment Modulation

Since exosomes contain lipids, proteins, and nucleic acids, they affect the tumor microenvironment [24, 53]. The tumor microenvironment consists of both cancerous cells and antigen‐presenting cells (APCs), including macrophages and B cells [51]. Exosomes with LMP1 contribute to the following processes after merging with the recipient cells: they induce immunosuppressive effects on NK cells, promote proliferation of LMP1‐negative cells by causing the transcription of IL‐8 in them, and finally, lead to epithelial–mesenchymal transition, which is highly associated with metastasis [44]. In addition, EBV latent infection is correlated with regulatory epigenome reprogramming, which facilitates EBV‐specific interactions between cancer cells and immune cells in the tumor microenvironment, resulting in immune suppression [54]. Therefore, regarding the immunosuppressive tumor microenvironment, dysfunctional CD8+ T cells produce high levels of immune checkpoint signaling molecules, such as programmed cell death protein‐1 (PD‐1) and its ligand PD‐L1. This weakened immune response aids NPC cells in escaping detection by the host’s immune system [47].

### 4.2. Autoimmune Diseases

#### 4.2.1. MS

##### 4.2.1.1. Potential Role of EBV Exosomes in Molecular Mimicry and Immune Dysregulation

In addition to malignancies discussed above, several autoimmune disorders are the consequence of EBV infection. MS can be correlated to EBV infection [55]. A wide range of patients with MS showed rising levels of antibodies against EBNA1 even before the appearance of neurological symptoms [56]. In EBV, the immune response is weakened. T cells play a key role in managing EBV, and in systemic autoimmune diseases, these T‐cell populations are often impaired or exhausted. As a result, persistent infections may occur along with ongoing cycles of relapses and remissions [55]. EBV exosomes stimulate proinflammatory response in APCs [56]. Inflammation can activate EBV‐infected B cells in lymphoid tissues, inducing the release of cytokines and EBERs, which can be secreted via exosomes, leading to inflammation that compromises the integrity of the blood–brain barrier. This disruption contributes to neuronal damage. Findings of elevated cytotoxic T lymphocyte activity targeting EBV during the early stages of MS show that anti‐EBV inflammatory responses can be associated with the development of MS [57]. EBV + B cells may lead to the worsening of MS plaques by sustaining inflammatory processes and molecular mimicry. Molecular mimicry occurs when a foreign peptide resembles a self‐peptide, causing the activation of autoreactive B and T cells. It has been discovered that there is a molecular mimicry between EBNA1 and the CNS protein GlialCAM1, which is known to be present in chronic‐active MS plaques [58].

#### 4.2.2. SLE

##### 4.2.2.1. EBV Exosomes and Their Contribution to Autoantibody Production

SLE is a complex disease related to reduced levels of complement proteins. Infection with EBV leads to the progression of SLE. In individuals with SLE, there is molecular mimicry between EBNA1 and cellular Ro 60 [55], and an autoimmune reaction may be induced by the molecular mimicry between SLE autoantigens and EBV antigens [59].

## 5. Diagnostic and Therapeutic Potential of EBV‐Derived Exosomes

### 5.1. Diagnostic

EBV is capable of vesicle formation and secretion, which is very effective on the tissue and tumor microenvironment. EBV possesses the ability to form and secrete vesicles, a process that significantly influences the tissue and tumor microenvironment. The presence of EBV exosomes in different body fluids suggests their potential utility as noninvasive biomarkers not only for diagnosis but also for prognosis [29]. Early diagnosis of the disease leads to better patient outcomes and simpler therapy. EBV‐related exosomes, which contain a considerable amount of pathogenic factors, can also be a strong target for cancer diagnosis and therapy. EBV‐derived vesicles contain various viral components, including proteins, RNAs, and microRNAs. Exosomal miRNAs and proteins exhibit significant potential as diagnostic tools for EBV‐associated diseases, offering advantages in sensitivity, specificity, and noninvasive detection compared to traditional methods [24].

Some of the most important proteins that are expressed in the most EBV‐related cancer cells are LMP1, LMP2A, and EBER. LMP1 and LMP2A are the key regulators of EBV to manipulate the host cells. Exosomes containing LMP1 can trigger tumor growth through activation of some signaling pathways, such as NF‐kB/PI3, which leads to the upregulation of antiapoptotic genes such as Bcl‐2. The exosomes containing LMP1 can inhibit T‐cell activation and proliferation, which results in EBV persistence in tumors such as NPC and Hodgkin’s lymphomas. Cyclophilin A (CYPA) is overexpressed in NPC exosomes and positively correlated with LMP1 levels. In MS, the levels of LMP2A increase in comparison with healthy cases. In addition, exosomal LMP2A may contribute to autoimmune response by enhancing B‐cell dysregulation and APCs [60]. Nevertheless, the diagnostic reliability of these biomarkers is debated and controversial. One of the concerning issues is the low sensitivity in clinical sample detection due to the scarcity of EBV‐positive exosomes. In addition, the major concern regarding the specificity of EBV exosome biomarkers is that LMP1 and BART miRNAs have occasionally been detected in noninfected derived exosomes.

In addition to these LMPs, exosomes secreted from EBV‐infected cells may contain EBERs, which are some noncoding RNAs consisting of 167 nucleotides with high expression in these extracellular vesicles. As a result, they are responsible for cellular transformation and antiviral innate immunity. The complex of EBERs and lupus antigen (La) ribonucleoprotein in the exosome can increase their stability, which leads to the alteration of recipient cell function and it could be used as a cancer diagnostic biomarker [24]. Furthermore, high levels of EBV miR‐BART1 and miR‐BART4 in gastric carcinoma have been observed. Not only proteins and microRNAs but also long noncoding RNAs are extensively studied as potential biomarkers. For example, in patients with NPC, high expression of LncRNA PVT1, in addition to miRNA‐155, has been detected. Exosomes from EBV‐infected cells act as potent modulators of the immune system by suppressing adaptive responses, activating innate immunity, and promoting viral persistence [5].

### 5.2. Therapeutic

One of the most important methods for disease therapy is drug delivery. Using nanoparticles such as liposomes is very common among these methods, although it has some limitations, such as low stability, synthesis, and cost. However, EVs have been extensively studied as a strong and suitable carrier in drug and gene delivery. Tumor‐derived exosomes play an essential role in proliferation and immune evasion. As a result, blocking these exosomes can enhance the immune system’s function to kill cancer cells. As an example, exosomes that contain galectin‐9 are responsible for inhibiting the EBV‐specific T‐cell proliferation and leading to apoptosis. Therefore, if we develop a treatment that blocks galectin‐9, the host immune system can be repaired. Instead of therapeutic targets, therapeutic delivery through exosomes can be replaced. Utilizing exosomes with anti‐EBV drugs might be a potent treatment that suppresses the tumor cell division and raises the stability of drug delivery more than other nanoparticles [61].

Exosomes are emerging as one of the promising tools for targeted therapy of EBV‐related diseases, especially tumors. Significant methods are applied to use these vesicles for targeted therapy, such as delivering therapeutic agents, disrupting mechanisms with the role of supporting tumors, and enhancing antitumor immunity. One of the examples of using engineered exosomes for targeting EBV is iRGD‐tagged exosomes loaded with antagomiR‐BART‐5p. This engineered exosome targets EBV‐encoded miR‐BART‐5p, which inhibits tumor vascularization in NPC patients. Utilizing iRGD tags will improve specificity and reduce off‐targets. Another strategy is using a kind of innate T‐cell‐like Vδ2‐T cells with antitumor potential against EBV. Exosomes containing Vδ2‐T (Vδ2‐T‐Exos) induce cell death through the FasL/TRAIL pathway and directly kill EBV‐associated tumors. In a mouse model, the implementation of this strategy resulted in a measurable decrease in tumor growth. [62]. Furthermore, the inhibition of the incorporation of EBV‐encoded molecules, such as LMP1, into exosomes or their subsequent release may serve to mitigate immune evasion and impede tumor progression [63]. Exosome‐based therapies have a number of advantages, including easier storage, lower toxicity, and reduced costs, but it is essential to optimize exosome engineering and their integration with immunotherapies in order to improve their efficacy. With all these pros, several drawbacks can make the usage of these vesicles challenging despite their application in targeted therapy. Large‐scale production of exosomes with consistent quality is very taxing. Exosomes can target a variety of cells. As a result, rising risks of off‐target effects are unavoidable. Even engineered exosomes can struggle to target the right place. Another thing that limits their clinical utility is low drug loading efficiency because exosomes often carry small amounts of agents such as antagomiRs and chemotherapeutics. Furthermore, isolating exosomes in the context of EBV research poses several challenges and has many complications [64]. Current techniques have this risk of isolating other EVs in addition to exosomes, complicating the study of EBV‐derived exosomes. Moreover, after isolation, analyzing exosome contents needs high‐tech and advanced devices, which can be costly. Achieving high‐purity exosome isolation is of high importance these days [32] (Table 2).

**TABLE 2:** Application of viral components in diagnosis and disease treatment.

Key component/target	Application	Findings	Sample type/detection methods
Galectin‐9	Therapeutic	Targeted for antiangiogenic and immune checkpoint modulation	Serum/ELISA
Vδ2‐T‐cell‐derived exosomes (FasL/TRAIL)	Therapeutic	Induce cell death and boost CD4+/CD8+ T‐cell responses	Mouse model/flow cytometry
Cyclophilin A (CYPA)	Diagnostic	Overexpressed in NPC exosomes	Plasma/ELISA
LMP1/LMP2A	Diagnostic	Multiple sclerosis (MS), autoimmune diseases, NPC, and Hodgkins lymphomas	Plasma, tissue/western blot, IHC
LncRNA PVT1 and miRNA‐155	Diagnostic	NPC	Plasma/RT‐qPCR

*Note:* NPC, nasopharyngeal carcinoma; CYPA, cyclophilin A.

Abbreviations: LMP, latent membrane protein; lncRNA, long noncoding RNA; MS, multiple sclerosis.

## 6. Challenges and Future Directions

As previously discussed, EBV‐derived exosomes have an essential role in intracellular communication, tumor formation, and progression. A major challenge specific to EBV exosome research is the poor sensitivity of biomarker detection in patient‐derived samples and the questionable specificity of candidates such as LMP1 and BART miRNAs, which have been reported in noninfected contexts. There are several challenges in the use of EVs in clinical trials, but the major hindrance is the lack of standardization in different isolation methods. The techniques for exosome isolation are mostly based on size, affinity, and precipitation, including ultracentrifugation, ultrafiltration, immunoaffinity, isolation kits, and microfluidics. However, these methods fail to isolate a completely pure exosome and lead to several complications in diagnostics and therapeutic applications.

Even though exosomes have potential as biomarkers, distinguishing EBV exosomes from normal exosomes and other EVs remains challenging. This difficulty is due to the complexity of body fluids. Different isolation protocols, such as ultracentrifugation, precipitation, and density gradient‐based methods, have been developed. These methods often fail to achieve high efficiency, which leads to co‐isolation of EBV exosomes and host‐derived EVs with similar composition and structure. However, some reports have mentioned that latent membrane proteins (LMP1–LMP2A) and some viral miRNAs, such as BARTs mentioned above, are unique EBV exosomal biomarkers. In contrast, some studies found that these candidate biomarkers can sometimes be found in vesicles from different infected or noninfected cells. As a result of incomplete isolation and lack of accuracy, the specificity of exosome targets will decrease, and off‐target effects will occur more frequently [61]. In order to overcome the current challenges, studies have to focus on highly sensitive molecular approaches such as digital‐PCR and single exosome analysis.

Different types of cells, such as tumor cells, stem cells, and mesenchymal stem cells, can secrete exosomes, load various molecules, and target a range of cells. Exosome modifications, such as loading miRNA, have become novel therapeutic strategies for cancer treatment in recent years. As an example, in EBV‐associated exosomes, miR‐BART10‐5p and hsa‐miR‐18a induce angiogenesis in the tumor microenvironment if they are overexpressed. Another investigation to improve the efficiency of therapy for EBV‐associated tumors is targeting EBV + exosomes, which is emerging as a promising strategy. The newer method is preventing EBV‐encoded molecules, which is more complex but more effective. However, the application of EBV + exosomes for therapeutic applications requires further studies and investigations [24].

## 7. Conclusion

EBV‐associated exosomes carry a diverse cargo of viral miRNAs and proteins that influence immune response and promote tumor progression. These features make them promising for noninvasive diagnosis and targeted therapy. On the diagnostic side, the urgent requirements include standardized protocols for exosome isolation, along with studies to confirm the reliability of biomarkers such as LMP1 and BART miRNAs. For therapeutic applications, important priorities stand out, including using engineered exosomes as drug delivery vehicles, preventing viral components from being packaged into exosomes, and testing exosome‐based vaccines in animal models of EBV‐associated disease in preclinical studies. Addressing these priorities is crucial for moving exosome‐based diagnostics and therapies from bench to bedside. Furthermore, a deeper understanding of the molecular mechanisms controlling selective loading of the cargo, intercellular communication, and the interaction between exosomes and recipient cells will be critical for the identification of novel therapeutic aims. Overall, continued interdisciplinary research connecting virology, immunology, bioengineering, and clinical investigation will be essential to translate the growing knowledge of EBV‐derived exosomes into effective diagnostic tools and therapies.

## Funding

The authors received no financial support for the research, authorship, and/or publication of this article.

## Conflicts of Interest

The authors declare no conflicts of interest.

## Data Availability

Data sharing is not applicable to this article as no datasets were generated or analyzed during the current study.
